# Epidemiological Cut-Off Values and Multidrug Resistance of *Escherichia coli* Isolated from Domesticated Poultry and Pigs Reared in Mwanza, Tanzania: A Cross-Section Study

**DOI:** 10.3390/ani12070835

**Published:** 2022-03-25

**Authors:** Conjester I. Mtemisika, Helmut Nyawale, Ronald J. Benju, Joseph M. Genchwere, Vitus Silago, Martha F. Mushi, Joseph Mwanga, Eveline Konje, Mariam M. Mirambo, Stephen E. Mshana

**Affiliations:** 1 Department of Microbiology and Immunology, Weill Bugando School of Medicine, Catholic University of Health and Allied Sciences, Mwanza P.O. Box 1464, Tanzania; helmutny@yahoo.com (H.N.); vsilago@bugando.ac.tz (V.S.); mushimartha@gmail.com (M.F.M.); mmmirambo@gmail.com (M.M.M.); stephen72mshana@gmail.com (S.E.M.); 2 Bugando Medical Centre, Molecular Biology Laboratory, Mwanza P.O. Box 1370, Tanzania; 3 Tanzania Veterinary Laboratory Agency, Ministry of Livestock and Fisheries, Mwanza P.O. Box 129, Tanzania; rbenju@yahoo.com (R.J.B.); jgenchwere@yahoo.com (J.M.G.); 4 Department of Epidemiology, Biostatistics and Behavioral Sciences, School of Public Health, Catholic University of Health and Allied Sciences, Mwanza P.O. Box 1464, Tanzania; jrmwanga@yahoo.co.uk (J.M.); ekonje28@yahoo.com (E.K.)

**Keywords:** antimicrobial resistance, epidemiological cut-off values, *Escherichia coli*, poultry, pigs

## Abstract

**Simple Summary:**

The objectives of this study were to determine the prevalence of multidrug resistance phenotypes and the distribution of *Escherichia coli* among poultry and pigs. Laboratory procedures were conducted according to standard operating procedures and international guidelines. Our findings showed that poultry and pigs reared in Mwanza, Tanzania, are colonized with resistant bacterial phenotypes. Further, different populations of intestinal flora, *E. coli*, exist between poultry and pigs.

**Abstract:**

Increasing antimicrobial resistance (AMR) colonizing domesticated animals is a global concern threatening food safety. This study aimed at determining the prevalence of multidrug resistance (MDR) and epidemiological cut-off values (ECVs) of *E. coli* isolated from poultry and pigs in Mwanza, Tanzania. This cross-sectional study was conducted between June and August 2021, involving 297 pigs, 191 broilers, and 203 layers. Rectal and cloacal swabs were collected and processed following standard guidelines. ECVs were determined using normalized resistance interpretation (NRI), a computer software, and descriptive analysis was performed using STATA version 13.0. The overall prevalence of MDR *E.* *coli* was 63.2%, whereas poultry (87.5% layers and 86.3% broilers) were more colonized than pigs (31.8%) (*p* < 0.001). Based on ECVs of antibiotics tested, *E. coli* from broilers, layers, and pigs exhibited different resistance patterns hence different populations. Exotic breed (*p* < 0.001) and recent antimicrobial use (*p* < 0.001) significantly predicted colonization with MDR *E. coli*. Veterinary officers should implement regulations that prohibit the inappropriate use of antimicrobial agents in livestock keeping.

## 1. Introduction

The use of antimicrobials in livestock to maintain health and promote production is increasing [1], resulting in antimicrobial selection pressure leading to the proliferation of antibiotic-resistant bacteria [2]. Generally, the use of antimicrobials in animals is reported higher in poultry and pigs than in cattle, threatening the safe consumption of poultry and pork and increasing environmental contamination with MDR bacterial strains [1]. Moreover, MDR strains may be transmitted to humans directly via contact with live animals or manure and indirectly via the consumption of contaminated animal products [3,4]. This may result in humans being colonized by multidrug-resistant (MDR) bacteria and subsequently MDR bacterial infections [3].

The increasing unregulated use of antimicrobials in livestock production, particularly in Tanzania, lacks AMR data to create evidence-based standard treatment guidelines for animals [5,6,7]. In Africa, including Tanzania, studies have documented antimicrobials use among domesticated animals ranging from 77% to 100%, whereas carriage of MDR producing bacteria, particularly Gram-negative bacteria were found to range from 20% to 100% [8,9]. The proportion of MDR strains among *E. coli* isolated from poultry and pigs was 55.2% and 44.8%, respectively, along the Msimbazi basin in Dar es Salaam, Tanzania [10]. However, human antimicrobial susceptibility testing disks and guidelines for interpretation of zones of inhibition used among animal surveillances lack veterinary breakpoints. This practice could over and/or under-report the burden of MDR in livestock [11].

In Mwanza, Tanzania, the prevalence of extended-spectrum β-lactamase (ESBL) among companion and domesticated farm animals was 21.7%. ESBL *E. coli* (93.3%) was predominantly isolated, and pigs were more colonized (33.1%) than other animals [8]. Despite the availability of this information, the prevalence and patterns of MDR Gram-negative bacteria (GNB), notably *E. coli,* is not clearly known as the previous study from the same setting used selective culture media to screen for ESBL producing GNB. The lack of this information may underestimate strategic efforts to prevent the emergence and spreading of MDR bacterial strains among livestock, humans, and environments. Therefore, we designed this study to investigate the prevalence and patterns of MDR *E. coli* and establish epidemiological cut-off values (ECVs) of *E. coli* colonizing poultry and pigs reared in Mwanza, Tanzania. The information from this study will not only facilitate the review of empirical treatment guidelines but also necessitate the implementation of MDR control and preventive measures among poultry and pigs reared in Mwanza, Tanzania. *E. coli* is frequently used as indicator bacteria to monitor trends of antimicrobial resistance (AMR) because it can easily acquire and preserve antibiotics resistance genes from other organisms in the environment and animal populations [12,13,14]. *E. coli* is also considered a good indicator of the selective pressure imposed by antimicrobial use (AMU) in food animals [12,15,16].

## 2. Material and Methods

### 2.1. Ethical Approval

Ethical approval for this study was obtained from the joint CUHAS/BMC research ethics and review committee with certificate number CREC/474/2021. Permission to conduct this study was requested from Livestock and Fisheries authorities. Livestock keepers (farmers) were requested to sign permission forms before sample collection. Unique identification laboratory numbers were used throughout the study.

### 2.2. Study Design, Population Setting, and Duration

This cross-sectional study was conducted between June and August 2021 among domesticated poultry and pigs reared in 16 wards located in 3 districts in Mwanza, Tanzania, namely, Nyamagana (Nyegezi, Buhongwa, Igoma, Busenga, Kilimahewa, Lumala, Mahina alliance, Malimbe, Mkolani, Nyamongolo, and Mabatini), Ilemela (Buswelu, Kiseke PPF, Nyamanoro, and Pasiansi), and Misungwi (Nyashishi).

### 2.3. Animals and Farms Selection

Pigs and poultry farms were systematically selected from a list provided by the livestock officers within the study area. Pig farms with pigs aged ≥ 20 weeks and poultry farms with ≥100 poultry were selected. A total of 29 farms (9-pig farms, 9-broiler farms, and 11-layer farms) were visited and enrolled in this study. Poultry farms were selected based on the number of flocks, and 5% of poultry ready to enter the food chain (aged ≥ 12 weeks) in each selected farm were identified for sampling.

For pigs, 10% of pigs per pig pen ready to enter the food chain (aged ≥ 20 weeks) in each selected farm were randomly identified and sampled. In all 9 farms, a total of 628 pigs were reared, and sampled pigs in each farm totaled: 1st farm 20 pigs, 2nd farm 31 pigs, 3rd farm 28 pigs, 4th farm 41 pigs, 5th farm 33 pigs, 6th farm 45 pigs, 7th farm 49 pigs, 8th farm 31 pigs, and 9th farm 19 pigs, a total of 297 pigs.

### 2.4. Sample Collection and Transportation

Fecal samples from the rectum (pigs) or cloaca (chicken) were collected using a sterile cotton swab. Briefly, a sterile swab was gently inserted into the cloaca/rectum and rotated to ensure sufficient sample was collected. Samples were transported to the Microbiology laboratory of the Catholic University of Health and Allied Sciences (CUHAS; Mwanza, Tanzania) in Stuart transport media in a clean, cool box within 8 h following collection.

### 2.5. Laboratory Procedure

#### 2.5.1. Isolation of *E. coli*

Swab samples were directly inoculated onto plain MacConkey agar (MCA; HiMedia, India followed by aerobic incubation at 37 °C for 18–24 h. After incubation, in the case of mixed growth, a single colony resembling *E. coli* among morphological predominant similar colonies (deep pinkish, round, mid-sized, and flat) was selected for the purity-plate, its sub-culture onto another plain MCA plate, which was incubated aerobically at 37 °C for 16–20 h, as reported previously [8,10]. Pure growth of presumptive *E. coli* was further identified by in-house prepared biochemical identification tests to species level.

#### 2.5.2. Physiological and Biochemical Identification of *E. coli*

The presumptive isolates of *E. coli* were preliminarily identified by using conventional in-house prepared physiological and biochemical identification tests, including lactose fermentation, production of CO_2_ from sugar fermentation, and sulfur production by triple sugar iron (TSI) agar; sulfur production, indole production, and motility by sulfur-indole-motility (SIM) medium; utilization of sodium citrate as the sole source of carbohydrate by Simmons citrate; and urease production by Christensen’s urea agar. Identification tests were interpreted as reported previously [17]. Identified isolates of *E. coli* were subjected to antibiotic susceptibility testing (AST) and phenotypic confirmation of ESBL production.

#### 2.5.3. Antibiotics Susceptibility Testing (AST)

All *E. coli* isolates were tested for antibiotics susceptibility by using the disk diffusion method as reported by Kirby-Bauer [18]. Briefly, isolates were suspended in sterile 0.85% normal saline and adjusted to 0.5 McFarland standard solution. Then, MHA plates were inoculated, and antibiotic disks were seeded within 15 min after inoculation of MHA plates. MHA plates were incubated aerobically at 37 °C for 16–18 h. The interpretations of zones of inhibitions were performed as recommended by the CLSI 29th Edition guidelines [19]. All *E. coli* that showed intermediate susceptibility to the antibiotics tested were regarded as resistant to such particular antibiotics. Antibiotics tested included ciprofloxacin (CIP 5 μg; HiMedia, Mumbai, India), ampicillin (AMP 10 μg; HiMedia, India), tetracycline (TE 30 μg; HiMedia, India), meropenem (MEM 10 μg; HiMedia, India), ceftazidime (CAZ 30 μg; HiMedia, India), gentamicin (CN 10 μg; HiMedia, India), cefepime (FEP 30 μg; HiMedia, India), and trimethoprim-sulfamethoxazole (SXT 25 μg; HiMedia, India).

#### 2.5.4. Screening and Phenotypic Confirmation of ESBL Production

Isolates from plain MCA were sub-cultured on MCA plates which were supplemented with cefotaxime 2 μg/mL (MCA-C) for the screening of ESBL producing *E. coli* (ESBL-EC) as documented previously [20]. Plates were incubated aerobically at 37 °C for 18–24 h. All isolates grown on MCA-C were further confirmed for ESBL production using the phenotypic method, a combination disc method recommended by the Clinical and Laboratory Standards Institute (CLSI) 29th Edition guidelines [19]. Briefly, bacterial suspensions in sterile normal saline equivalent to 0.5 McFarland standard solution were prepared and inoculated on the entire surfaces of Mueller Hinton agar (MHA; HiMedia, India). Then, disks of ceftazidime 30 μg (CAZ 30 μg; HiMedia, India) with and without clavulanic acid 10 μg (CAZ/CA 30/10 μg; HiMedia, India) were seeded on inoculated MHA plates and incubated aerobically at 37 °C for 16–18 h. Isolate exhibiting a difference of ≥5 mm zone of inhibition between CAZ 30 μg and CAZ/CA 30/10 μg were phenotypically confirmed as ESBL-EC.

#### 2.5.5. Quality Control

*E. coli* ATCC 25,922 and *E. coli* ATCC 35,218 were used as control strains to control the performance of culture media, incubation conditions, and antibiotic disks.

### 2.6. Data Management and Analysis

Data were entered into Microsoft Excel for cleaning and coding, then into STATA version 13.0 for analysis and NRI computer software, where calculations were performed to define wild type populations by establishing ECVs. All isolates that showed resistance to one or more antibiotic agents in at least three classes were considered multidrug-resistant (MDR) strains. Continuous data were presented as mean (±standard deviation: SD)/median (interquartile range: IQR), whereby categorical data were presented as percentages. Chi square analysis was used to show the association between outcome (i.e., MDR colonization) and variables (i.e., antimicrobial exposure, breed of livestock, and species). A *p*-value of <0.05 was considered statistically significant. Epidemiological cut-off values were determined by computer software called Normalized resistance interpretation (NRI; Bioscand AB, Täby, Sweden, International Patent Application WO 02/083935 A1). This method analyzes inhibition zone diameters produced from the disk diffusion technique of antimicrobial susceptibility testing. The NRI software produced a histogram that showed the ECVs and distribution of wild type (sensitive isolates) and non-wild type (resistant isolates) bacteria and the number of SD from the mean [21,22]. In some circumstances where the obtained ECVs were very low, i.e., a zero or negative number, due to the high resistance of *E. coli* to a particular antibiotic, mean was used as a tentative ECV estimate as reported elsewhere [11,23].

## 3. Results

### 3.1. Characteristics of and Antimicrobials Use among Livestock Enrolled in the Study

A total of 691 livestock, including 27.6% (191/691) broilers, 29.4% (203/691) layers, and 42.9% (297/691) pigs, were sampled from 29 livestock keepers. The majority of livestock were exotic—71.4% (493/691). The recent date of antimicrobial use (AMU) was not known 62.8% (434/691); however, the majority of livestock were administered antimicrobials for therapeutic reasons 86.4% (597/691). About 41.1% (284/691) of livestock were on antimicrobials prescribed by a Veterinary officer (Table 1)

### 3.2. Commonly Used Classes of Antibiotics among Livestock Enrolled in this Study

It was observed from this study that antibiotic agents such as tetracycline, sulfonamides, and quinolones were commonly used in poultry keeping. However, in pigs, antibiotic agents in tetracycline and sulfonamides were common, although classes of quinolones, macrolides, and aminoglycosides were not reported to be used in pigs in this study (Table 2).

### 3.3. Culture Results

**Figure 1:**
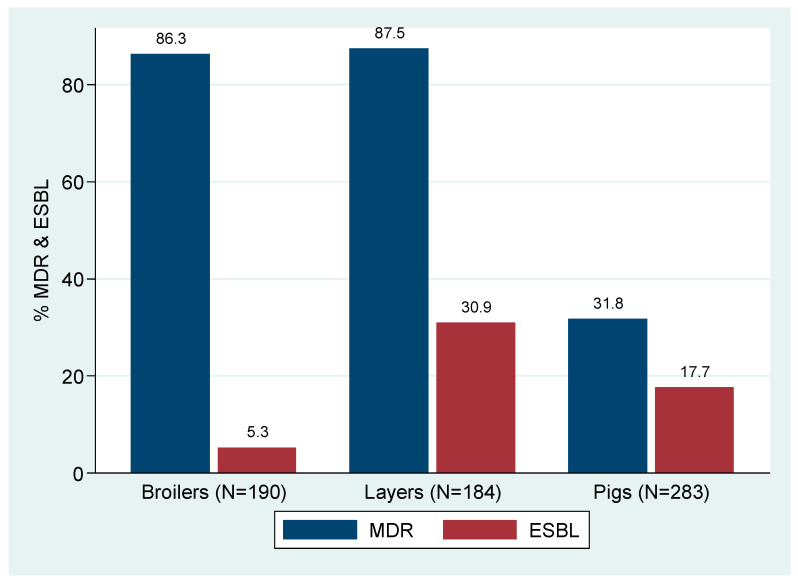
Percentages of MDR and ESBL *E. coli* from broilers, layers, and pigs.

A total of 95.1% (657/691) *E. coli* were isolated from pigs and poultry, of which a total of 63.2% (415/657) were MDR *E. coli*. The MDR proportions in broilers, layers, and pigs were 86.3% (164/190), 87.5% (161/184), and 31.8% (90/283), respectively. A total of 17.8% (117/657) screened positive for potential ESBL production by MCA-C plates. Furthermore, all presumptive ESBL producing *E. coli* 100% (117/117) were phenotypically confirmed to be ESBL producers. ESBL production was significantly high among *E. coli* from layers (30.9%, 57/184) compared to pigs (17.7%, 50/283) and broilers (5.3%, 10/190) *p* < 0.001) (Figure 1).

### 3.4. Resistance Patterns of E. coli to Antibiotics Tested

Percentage resistance for ciprofloxacin, tetracycline, ampicillin, and trimethoprim/sulfamethoxazole, was high among *E. coli* isolated from poultry compared to pigs. However, tetracycline was the most resistant antibiotic among *E. coli* from pigs compared to other antibiotics tested (Table 3).

### 3.5. ECVs of Tested Antibiotics against E. coli

**Figure 2:**
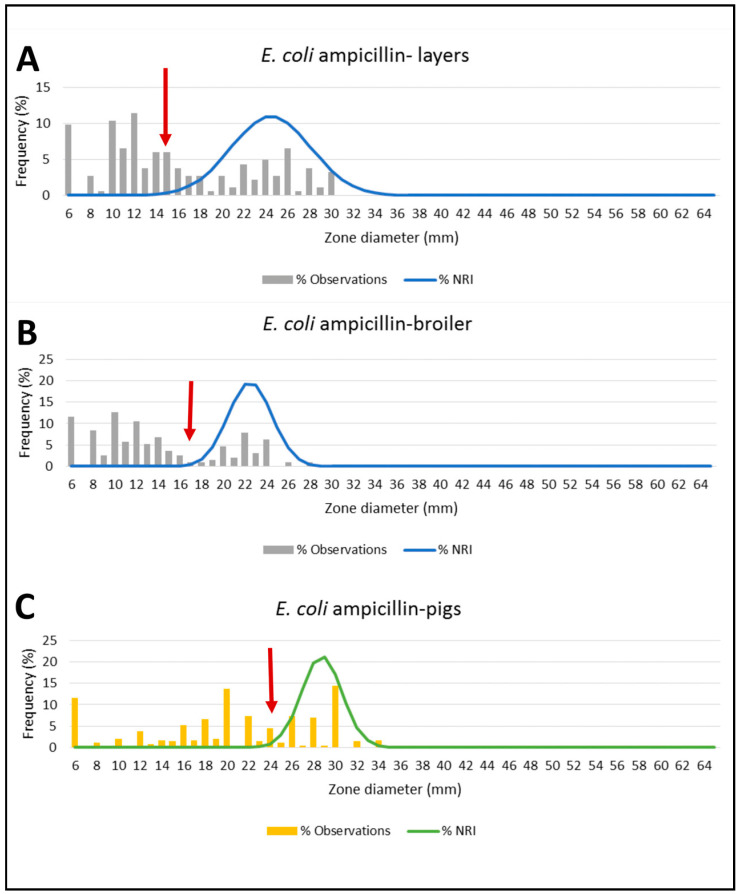
The ECVs of AMP tested against *E. coli* isolated from layers, broilers, and pigs. The arrow indicates where the ECV is located in a histogram, and distribution of WT and non-WT *E. coli* as determined by NRI. Subfigure (**A**) shows ECV of AMP tested against *E. coli* from layers which is 15 mm; subfigure (**B**) shows ECV of AMP tested against *E. coli* from broilers which is 17 mm; and subfigure (**C**) shows ECV of AMP tested against *E. coli* from pigs which is 24 mm.

The ECV of antibiotics tested against *E. coli* isolated from broilers ranged from 11 mm for TET to 18 mm for FEP and CIP; in layers, it ranges from 9 mm for TET to 18 mm for MEM; and from 9 mm for TET to 30 mm for SXT in pigs. In general, *E. coli* from poultry and pigs exhibited different resistance patterns to antibiotics tested and, therefore, ECVs. This observation indicates that *E. coli* from poultry and pigs belong to different populations, whereby *E. coli* from pigs were more susceptible to antibiotics tested than *E. coli* from poultry (Table 4 & Figure 2).

There is a varying proportion of wild type (WT) distribution when a human clinical breakpoint is used compared to when ECVs are used. Notably, clinical breakpoints under-report percentages of WT-*E. coli* for CIP, TE, MEM, FEP, and CAZ in poultry, whereas clinical breakpoints over-report percentages of WT *E. coli* for AMP, CN, and SXT in pigs (Table 5).

### 3.6. Factors Associated with MDR Colonization

The chi square test showed poultry (broilers and layers) are significantly colonized with MDR *E. coli* (*p* < 0.001), furthermore, exotic breeds were significantly colonized with MDR *E. coli* (*p* < 0.001), and the recency of the antimicrobial use predicts the colonization of MDR (*p* < 0.001) (Table 6).

## 4. Discussion

Our findings showed different levels of resistance and different ECVs to commonly used antimicrobials in livestock among *E. coli* isolated from poultry and pigs. Most of the animals enrolled in this study (95.1%) were colonized with *E. coli,* similar to a study by Kimera et al., which observed colonization in 80.5% of animals [10] The majority of livestock enrolled were exotic breeds (71.4%) compared to a study done by Seni et al., who enrolled more local breeds (66.7%) [8]. Furthermore, exotic breeds were exposed to antimicrobial use more than local breeds. Generally, about two-thirds (63.2%) of *E*. *coli* isolated from livestock in our study were MDR, similar to a study done in Morogoro, which reported a prevalence of 65.1% [24]. A study done in Dar es Salaam reported low MDR prevalence (51.6%), which is lower than in the current study [10] The high prevalence of MDR in our study might be due to variations in farming conditions and antibiotic use. Furthermore, the level of MDR in the present study is lower than what was observed in China (83%) [25]. This might be explained by the fact that there is different antimicrobial exposure time and frequency, and China is one of the largest users of livestock antimicrobials in the world, increasing the antimicrobials selection pressure as a result of high MDR prevalence [25,26].

It was observed that over three-quarters and one-third of *E. coli* isolated from poultry and pigs were MDR, respectively. As documented previously [10] the level of MDR was significantly higher in poultry (in broilers and layers) than in pigs because there is a higher antimicrobial use in poultry keeping than in keeping pigs, as observed in this and other studies [24,27,28].

We observed more resistance to antibiotics of class quinolones, penicillin, tetracycline, and sulfonamides. High resistance to antibiotics of these classes was not surprising because these were the antibiotics reported to be commonly used by the livestock keepers in this study. MDR patterns observed in this study are in line with what was previously reported in Tanzania (quinolones, penicillin, tetracycline, and sulfonamides), China (tetracycline, sulfonamides, penicillin, quinolones), and Nigeria (tetracycline, sulfonamides, penicillin) [7,10,25,29].

ESBL production is one of the commonest MDR phenotypes. In this study, it was observed that about one-fifth of *E. coli* isolates from poultry and pigs were ESBL producers. Similar to a previous study done in the same region, Mwanza, Tanzania, reported a prevalence of 21.7% [8]. However, the proportion of ESBL producing *E. coli* in our study is lower than in a study done in another region in Tanzania, which reported a prevalence of 65.3% [10]. This might be due to different exposure times to beta-lactam and cephalosporin in livestock keeping, and different *E. coli* populations in different geographic locations.

ECVs determined from NRI are based on the assumption that *E. coli* isolated from broilers, layers, and pigs carrying resistance mechanism/non-wild type exhibit smaller inhibition zone diameters (IZD) than *E. coli* without resistance mechanism/wild type. To the best of our knowledge, this is the first study in Mwanza, Tanzania, to report the ECVs to the commonly used antibiotics tested against *E. coli* isolated from poultry and pigs.

According to this study, the majority of *E. coli* from poultry and pigs were wild type meropenem, ceftazidime, cefepime, and gentamicin. This could be explained by the fact that these antibiotics are not readily available over the counter; that they are expensive (meropenem), while the gentamicin, ceftazidime, and cefepime mode of administration is intravenous (IV), which is not easy for the livestock keepers. This is further supported by the fact that livestock keepers did not report using these antibiotics.

The majority of *E. coli* from broilers were non-wild type to ciprofloxacin, trimethoprim-sulfamethoxazole, and ampicillin, while the majority of *E. coli* from non-wild type layers to tetracycline and trimethoprim-sulfamethoxazole. The majority of *E. coli* from pigs were non-wild type to ampicillin. This is not surprising since these antibiotics are commonly used, readily available over the counter, and the cheapest [9]. Furthermore, most livestock keepers from this study reported commonly using antibiotics from tetracycline, quinolones, sulfonamides categories.

A study in China reported 92.3% of *E. coli* from chicken respiratory tract infections were non-wild type to danofloxacin (quinolones), while 22.3% were non-wild type to apramycin (aminoglycosides). Similar observations were seen in this study in *E. coli* from broilers, where the majority were non-wild type to ciprofloxacin (quinolones), and the majority of *E. coli* from poultry were wild type to gentamicin (aminoglycosides) [27]. A study done by Yang et al. reported ECV of danofloxacin (quinolones) tested against *E. coli* from pigs to be 8 μg/mL using MIC [28].

However, contrary to poultry, most *E. coli* from pigs were wild type to most antibiotics tested. This can be explained by the fact that pigs are not exposed to high antimicrobial use compared to poultry. Furthermore, some antibiotics reported to be used in poultry were not reported to be used in pigs in this study, such as quinolones, macrolides, and aminoglycosides.

This study observed different ECVs to the same antibiotics tested to *E. coli* from poultry and pigs, meaning different *E. coli* population, this is not astonishing since poultry and pigs are exposed to different antibiotics with different frequencies of use. Contrary to what was observed in Sweden, the wild type distribution of *E. coli* isolated from human and wild birds in the same area was identical [23].

This study observed that human clinical breakpoints (CLSI) could lead to both over and under-reporting antimicrobial resistance burdens. Similar observations were made by Doidge et al. [11] in the UK, although it was in sheep and beef.

MDR colonization can be attributed to different factors. The most common is antimicrobial pressure which causes the selection of resistant bacteria. In this study, it was observed that exotic breeds were found to predict MDR colonization among poultry and pigs, and there was a significant association of MDR colonization to broilers and layers. This is not surprising as it might be contributed by the fact that there is high antimicrobial use in exotic breeds and poultry keeping. A study done by Seni et al. observed that exotic breeds were more at risk of being colonized with ESBL bacteria than local breeds [8]. ESBL is one of the common MDR phenotypes. Similar to what was observed in the current study, it was observed in a study done by Nonga et al., Katakweba et al., and Kimera et al. that poultry farming is associated with uncontrolled use of both veterinary and human antimicrobials [5,24,28]. A systematic review by Mshana et al. also reported that poultry production accounts for high antimicrobial use in Africa [7]. Recent antimicrobial use was found to predict MDR colonization in the present study. This can be explained by the fact that antimicrobial use creates selection pressure allowing resistant bacteria to multiply and propagate. It was reported that uncontrolled use of antimicrobials in livestock keepings as a growth factor, prophylaxis, and/or therapeutics had been associated with the emergence of MDR bacteria [29].

## 5. Conclusions

*E. coli* from layers and broilers are more resistant to commonly used antibiotics than *E. coli* isolates from pigs. Distinct populations of *E. coli* were circulating in layers, broilers, and pigs based on ECVs of different antibiotics tested, which was likely due to differences in antibiotic exposure and breeding type. Furthermore, recent antimicrobial use and exotic breeds predicted MDR colonization which might result from high antimicrobial use. Further studies involving other laboratories are needed to establish the ECVs for commonly used antibiotics and the data used to monitor resistance and further research to establish veterinary breakpoints. Veterinary officers should implement regulations that prohibit the inappropriate use of antimicrobial agents in livestock keeping. More studies to establish the genotypes of *E. coli* circulating in these species are warranted to provide data to monitor the emergence of new *E. coli* strains/genotypes.

## 6. Study Limitation

Limited knowledge of livestock keepers on antimicrobial used in their livestock may impact statistical analysis, particularly associated with ESBL carriage. In addition, the ECVs data are from a single laboratory, and, therefore, should be carefully interpreted to reflect the local settings. The resistance frequency may be underestimated, as only one colony per sample was analyzed.

## Figures and Tables

**Table 1:** Characteristics and AMU of livestock.

Variables		Frequency (*n*)	Percentage (%)
Livestock	Broiler	191	27.6
	Layers	203	29.4
	Pigs	297	42.9
Breed	Exotic	493	71.4
	Local	198	28.7
Recent antimicrobial date	2 months ago	48	6.9
	1 month ago	30	4.3
	2 weeks ago	100	14.5
	1 week ago	79	11.4
	Not known	434	62.8
Purpose of antimicrobial use	Prophylaxis and Therapeutic	94	13.6
	Therapeutic	597	86.4
Antimicrobial prescription	Agro vet shop/Vet shop	60	8.7
	Another farmer	45	6.5
	Myself/family member	142	20.6
	Paraveterinarian	115	16.6
	Paraveterinarian/Vet shop	25	3.6
	Veterinary officer	284	41.1
	Vet officer/myself/family member	20	2.9

**Table 2:** Antimicrobial classes commonly used in livestock keeping.

Antimicrobial Class	Poultry		Pigs	
	Frequency (*n*)	Percentage (%)	Frequency (*n*)	Percentage (%)
Tetracycline, sulfonamides	121	30.72	161	54.2
Tetracycline, quinolones	202	51.29		
Tetracycline, sulfonamides, macrolides	20	5.08	-	-
Tetracycline, macrolides	10	2.54	-	-
Tetracycline, aminoglycosides	21	5.33	-	-
Quinolones, aminoglycosides	20	5.08	-	-
Not known	-	-	136	45.8

**Table 3:** Percentage susceptibility of *E. coli* to antibiotic agents tested.

Antibiotics	Broilers (*n* = 190)	Layers (*n* = 184)	Pigs (*n* = 283)	*p* Value
	R	R	R	
**CIP**	180 (94.7%)	165 (89.7%)	67 (23.7%)	0.001
**AMP**	134 (70.5%)	115 (62.5%)	80 (28.2%)	0.001
**MEM**	6 (3.2%)	11 (6%)	37 (13.1%)	0.001
**TET**	166 (87.4%)	165 (89.7%)	140 (49.5%)	0.001
**CAZ**	36 (18.9%)	80 (43.5%)	76 (26.9%)	0.001
**SXT**	131 (68.9%)	161 (87.5%)	63 (22.3%)	0.001
**CN**	44 (23.2%)	41 (22.3%)	30 (10.6%)	0.001
**FEP**	35 (18.4%)	71 (38.6%)	69 (24.4%)	0.001

**Key:** CIP, ciprofloxacin; AMP, ampicillin; MEM, meropenem; TET, tetracycline; CAZ, ceftazidime; SXT, trimethoprim/sulfamethoxazole; CN, gentamicin; FEP, cefepime.

**Table 4:** ECVs of tested antibiotics against *E. coli*.

Antimicrobials	Disk Content	Broiler ECVs	SD	Layer ECVs	SD	Pigs ECVs	SD
CIP	5 μg	18 *	5.55	17	2.29	22	4.18
TET	30 μg	11 *	4.13	9 *	9.14	9	4.65
AMP	10 μg	17	2.00	15	3.62	24	1.85
MEM	10 μg	16	3.94	18	4.29	18	5.25
CAZ	30 μg	15	3.15	10	4.39	12	4.08
FEP	30 μg	18	3.26	11	5.19	18	4.36
CN	30 μg	15 *	3.04	10	2.95	16	2.84
SXT	1.25/23.75 μg	15 *	6.41	12 *	4.63	30	2.97

**Key:** CIP, ciprofloxacin; AMP, ampicillin; MEM, meropenem; TET, tetracycline; CAZ, ceftazidime; SXT, trimethoprim/sulfamethoxazole; CN, gentamicin; FEP, cefepime. * Mean used as ECV tentative estimate. SD = standard deviation.

**Table 5:** Comparison of ECVs and CLSI clinical breakpoints of antibiotics tested against *E. coli* isolated from poultry and pigs.

Antimicrobial Agents	Broilers: ECVs (%WT)	Broilers: CBs (%S)	Layers: ECVs (%WT)	Layers: CBs (%S)	Pigs: ECVs(%WT)	Pigs: CBs (%S)
CIP	31.1	5.3	51.1	10.3	84.5	76.3
TE	51.6	12.6	48.4	10.3	70.3	50.5
AMP	29.5	29.5	57.6	37.5	38.5	71.7
MEM	100	96.8	96.2	94.02	98.6	86.9
CAZ	99.5	81.1	95.7	56.5	97.2	73.1
FEP	100	81.6	97.3	61.4	96.8	75.3
CN	77.4	76.8	95.1	77.7	83.7	89.4
SXT	36.8	31.1	41.3	12.5	63.3	77.7

Key: CIP = ciprofloxacin; TE = tetracycline; AMP = ampicillin; MEM = meropenem; CAZ = ceftazidime; FEP = cefepime; CN = gentamicin; SXT = trimethoprim-sulfamethoxazole; ECVs = epidemiological cutoff values; WT = wild type as per ECVs; CBs = clinical breakpoints; and S = susceptible as per clinical breakpoints.

**Table 6:** Factors associated with MDR colonization.

Variables		MDR		CHI ANALYSIS	
		Positive *n* (%)	Negative *n* (%)	χ^2^	*p* Value
**Livestock**	Broiler	164 (86.3)	26 (13.7)	210.24	<0.001
	Layers	161 (87.5)	23 (12.5)		
	Pigs	90 (31.8)	193 (68.2)		
**Breed**	Exotic	353 (75.6)	114 (24.4)	107.11	<0.001
	Local	62 (32.6)	128 (67.4)		
**Recent antimicrobial use**	2 months ago	22 (52.4)	20 (47.6)	153.51	<0.001
	1 month ago	22 (78.6)	6 (21.4)		
	2 weeks ago	89 (91.8)	8 (8.3)		
	1 week ago	68 (86.1)	11 (13.9)		
	Not known	214 (52.1)	197 (47.9)		

## Data Availability

The data presented in this study are available on request from the corresponding author.
